# The Regentime stem cell procedure efficacy in asthma treatment: A case report

**DOI:** 10.1097/MD.0000000000042887

**Published:** 2025-06-13

**Authors:** Rita T. Boulos, Cynthia F. Najjoum, Vanessa J. Mansour, Elsa A. El Asmar, Nassim H. Abi Chahine

**Affiliations:** a Regentime Clinic, Beirut, Lebanon.

**Keywords:** asthma, case report, lung regeneration, stem cell therapy, the Regentime procedure

## Abstract

**Rationale:**

Asthma is a chronic respiratory condition affecting millions worldwide. It is characterized by airway inflammation leading to airflow obstruction and bronchial hyperresponsiveness. Despite optimal pharmacological treatment, some people fail to achieve full control of their symptoms and suffer from recurrent debilitating asthma attacks.

**Patient concerns:**

We report the case of a 54-year-old female patient who originally presented for stem cell treatment of her ankle. She complained of chronic left ankle pain and swelling that worsened with weight-bearing activities. During her medical history taking, she described experiencing dyspnea and wheezing both during the daytime and on more than 1 night weekly.

**Diagnoses:**

She had an obstructive pattern on spirometry and her symptoms were consistent with the diagnosis of moderate persistent asthma. She was also previously diagnosed with osteoarthritis affecting her tibiotalar and talocalcaneal joints.

**Interventions:**

The patient received partially differentiated autologous stem cells in her affected joints. Another portion of stem cells was treated and administered to her via inhalation according to the Regentime protocol for lung regeneration.

**Outcomes:**

Upon regular follow-up, the patient reported gradual improvement of her osteoarthritis symptoms, reaching full recovery of the ankle within 5 months following treatment. She also noted the progressive amelioration of her asthma manifestations until their disappearance during the first month after stem cell administration. Her clinical remission was maintained for at least 24 months with ongoing follow-up to date and progressive improvement on physical examination.

**Lessons:**

The successful outcome of this case marks the potential of the Regentime stem cell protocol for lung regeneration as a promising therapeutic approach for managing chronic respiratory conditions such as asthma.

## 1. Introduction

Asthma is a chronic respiratory condition characterized by episodic airway inflammation and bronchoconstriction leading to wheezing, coughing, and chest tightness.^[1]^ In 1860, Henry Hyde Salter described asthma as paroxysmal episodes of dyspnea with periods of normal breathing patterns in between. Thirty years later, Paul Ehrlich described aniline staining patterns for eosinophils and mast cells in the asthmatic sputum. He also suggested black coffee as a bronchospasm treatment due to its theobromine content, a theophylline derivative. Thus, toward the end of the 19th century, asthma was perceived as a distinct pathological condition with characteristic etiology, manifestations, and treatment.^[2]^

Asthma is more common in boys during childhood and until puberty when the male-to-female ratio equalizes. During adulthood, the prevalence becomes higher in females.^[3]^ Several genetic and environmental risk factors are implicated in increasing the risk of developing asthma. Genes that have been shown to play a role include the beta2 adrenergic gene, genes involved in T helper 1 and T helper 2 cell differentiation, as well as genes implicated in the cellular responses related to atopic diseases.^[4]^ Environmental risk factors include active and passive smoking, air pollution, obesity, occupational contributors, and infections.^[4]^ The classical asthma symptoms include expiratory wheezing, coughing that is worse at night, shortness of breath, and chest tightness. This pattern of respiratory symptoms occurs and varies in intensity following exposure to environmental triggers, exercise, and infections.^[5]^

Pharmacologic treatment is the mainstay of chronic asthma management in most patients. It is started depending on the stage of the condition (mild intermittent, mild persistent, moderate persistent, and severe persistent) and then escalated or de-escalated according to the response to treatment. Medications include a combination of inhaled corticosteroids, short-acting beta-agonists, long-acting beta-agonists, muscarinic antagonists, antileukotrienes, anti-IgE, and anti-interleukin-5 (anti-IL-5) depending on the classification of asthma severity.^[6]^ Some people fail to achieve full control of their symptoms despite adherence to medications, including costly biological agents.^[7]^ There are several hypotheses regarding the pathological mechanisms behind refractory asthma in such patients. These include markedly increased local eosinophils and Th2-driven cytokines that are unresponsive to corticosteroids, elevated levels of neutrophils compared with those of patients with mild asthma, which can significantly impact the structure and function of the lungs, the development of irreversible structural remodeling of the airways, and the extension of the inflammation to small distal airways.^[8]^

A new treatment modality that addresses the unmet needs of patients suffering from asthma, namely the refractory type, is therefore essential to explore. A novel experimental study demonstrates the efficacy of stem cells’ secretions in asthma treatment in mice. In fact, the newly described migrasomes, which are extracellular vesicles (EVs) that can be secreted by mesenchymal stem cells (MSCs), are shown to interfere in the pathogenic mechanisms of asthma by inhibiting dendritic cells (DCs) activation, reducing Th2 cytokines, and significantly decreasing airway inflammation symptoms in asthmatic mice.^[9]^ In addition, the efficacy of allogeneic MSCs administration to animal models has been suggested due to their potential to directly inhibit Th2 cytokines profile^[7]^ and improve airway remodeling as opposed to previous speculations about their differentiation into fibroblasts triggering asthma progression.^[10]^ Building on this, our report is the first to highlight the Regentime partially differentiated autologous bone marrow-derived stem cell procedure as a promising potential novel treatment option for asthmatic patients.

## 2. Case presentation

A 54-year-old female known to have moderate persistent asthma for 7 years presented to the Regentime research clinic seeking stem cell therapy for an osteochondral lesion of her talus bone (left talar dome). She complained of ankle pain increasing with weight-bearing activity. She also had notable ankle joint swelling, stiffness, locking, and catching. She had a history of seasonal allergies and 30-pack-year smoking. No significant family medical history was noted. She reported having episodes of dry cough, wheezing, and dyspnea daily during the daytime and for more than 1 night per week. Her medications included a chronic low dose of inhaled corticosteroids, muscarinic antagonists, and short-acting beta-agonists when needed. Upon physical examination, there was prominent wheezing, prolonged expiration, tachypnea, and hyperresonance.

### 2.1. Methods

**Figure 1.**
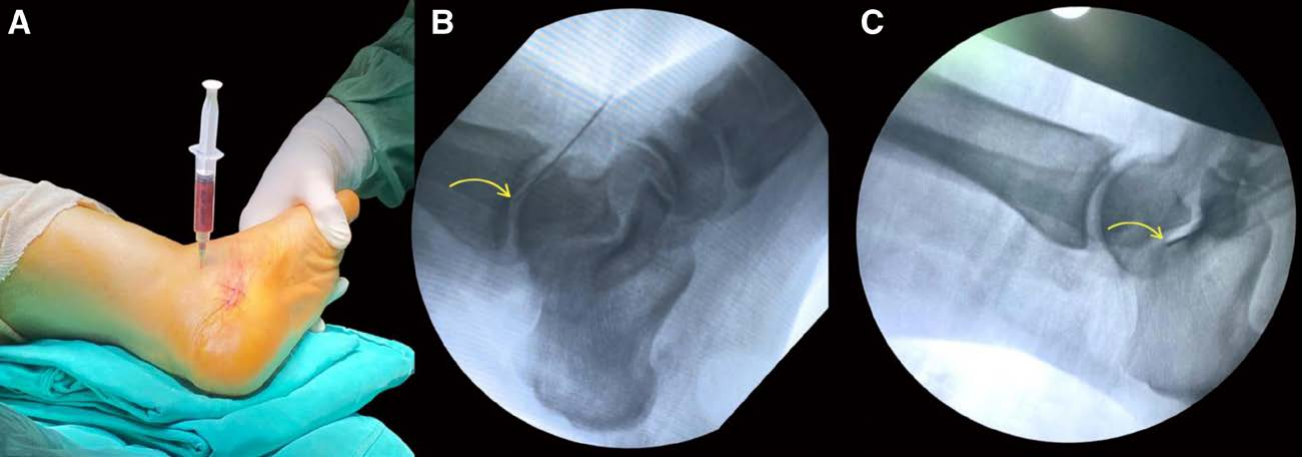
Tibiotalar and talocalcaneal injections of stem cells postincubation with cartilage RDA. (A) The tibiotalar injection, (B) C-arm image with the arrow showing the needle tip during the stem cell injection in the tibiotalar joint, and (C) C-arm image with the arrow showing the needle tip during the stem cell injection in the talocalcaneal joint. RDA = Regentime differentiating agent.

The patient decided to undergo the Regentime stem cell procedure for her ankle. Simultaneously, she was proposed a trial of a newly developed lung regeneration protocol, on which she agreed, and signed an adequate consent form. She was administered granulocyte colony-stimulating factor injections in a weight-adjusted dosage, after which a rise in her white blood cells was registered. A bone marrow aspirate was then obtained from her posterior superior iliac crests using heparinized aspiration syringes. The aspirate was incubated for 12 hours. Following incubation, mechanical purification using a 1500 G-Force centrifuge was performed, for the cellular buffy coat to be collected. Stem cells were equally divided into 2 portions, A and B. Both portions were incubated for 12 hours at 37°C with different Regentime differentiating agents (RDAs), consisting respectively of a cartilage RDA for portion A and a lung RDA for portion B. It is noteworthy that RDAs derive from tissue-specific sheep peptides. After 12 hours, the final product was maintained on a 3-D shaker for 20 minutes. In the operating room, under a C-arm X-ray, the patient underwent a direct injection of half of the portion A (treated with the cartilage xeno-ultrafiltrate) in her left tibiotalar joint (Fig. 1A, B). The other half was injected into her left talocalcaneal joint (Fig. 1C). Postoperatively, in her regular room, she received portion B (treated with the lung xeno-ultra-filtrate) via inhalation and intravenously. Before administration, portion B was divided into 2 equal parts of 9 mL each, as our clinic registered 15 mL of stem cells and 3 mL of added lung RDA. The first part was inhaled over 3 sessions, each consisting of 3 mL cellular product with 5 mL normal saline 0.9%, separated by 2-hour intervals. The second part was administered intravenously after being mixed with 500 mL of normal saline 0.9% over 6 hours. During the infusion, light compression was sustained at the tibiotalar site of injection and 1 g of paracetamol was administered. While under monitoring the patient experienced no undesirable effects for the next 24 hours.

### 2.2. Results

After treatment, monthly follow-up was scheduled via phone calls as the patient resided abroad and faced financial constraints. She had 2 in-person follow-up visits at 4- and 12-month posttransplantation. She reported progressive amelioration of her left ankle symptoms leading to total recovery during the 5th month of follow-up. As for her respiratory symptoms, the patient noted cardinal amelioration 2 days following the transplantation. At 1-month posttransplant, she informed the follow-up team of the total absence of any asthma symptoms, along with no further need for asthma medications. However, she declined further objective testing, expressing no desire for additional evaluations while continuously feeling better. Her physical examination 4 months posttreatment revealed normal breath sounds, no wheezing, no tachypnea, and mild residual swelling of the left ankle but with a normal range of motion. At the 12-month in-person follow-up, she had a normal physical examination with clear lung auscultation and normal left ankle range of motion without any swelling. During the 24-month follow-up monthly calls, the patient maintained complete clinical remission of her symptoms, with no reported changes in smoking habits, dietary changes, physical activity, or other medication use.

## 3. Discussion and conclusions

Asthma is a chronic inflammatory airway condition involving bronchial hyperresponsiveness and airflow obstruction. Its prevalence is increasing at a rapid rate, posing a significant global health concern.^[4]^ Interventions include avoidance of triggers, controller medications, and rescue medications.^[5]^ A large portion of asthma patients lead a normal life with limited restrictions given that they adhere to chronic and/or rescue medications. On the other hand, despite adequate treatment, a significant portion of patients report poorly controlled symptoms resulting in a lower quality of life and, therefore, increased healthcare costs.^[11]^ Our patient with moderate persistent asthma reports complete clinical recovery of her symptoms with no further need for asthma medications and no side effects observed following the Regentime stem cell treatment.

Inflammation plays a key role in the pathophysiology of asthma. The process begins with exposure to an irritant or an allergen leading to the interaction between multiple immune cells and mediators within the airways and resulting in the characteristic pathophysiological asthma features. DCs residing at the epithelial surfaces of the airways initiate the cascade by interacting with inflammatory cells in regional lymph nodes and stimulating Th2 polarization.^[2,12]^ The Th2 cytokine repertoire includes interleukin-4 (IL-4), which plays a role in Th2 cell differentiation,^[12]^ IL-5 and granulocyte-macrophage colony-stimulating factor, which synergistically recruit eosinophils and amplify their differentiation and survival,^[13,14]^ IL-13 which signals the shift from IgM to IgE that participates in the release of mediators from mast cells causing bronchoconstriction, and IL-1β and tumor necrosis factor-α both playing a role in the amplification of the inflammation.^[12,13]^ In addition, there is a reduction in the regulatory T cell (Treg) population along with its associated functional defects and inadequate differentiation. Tregs normally suppress T effector cells via several immunosuppressive mechanisms involving IL-10 and transforming growth factor-β; therefore, a defect in Treg numbers and immunomodulatory mechanisms plays a key role in asthma pathogenesis.^[15]^ Furthermore, macrophages constitute around 70% of immune cells in the lungs and have a heterogeneous spectrum of inflammatory and anti-inflammatory functions. The M1 macrophage subtype is pro-inflammatory, while the M2 subtypes (M2a, M2b, and M2c) are generally mediators of allergic reactions and participate in fibrosis and tissue remodeling. In asthmatic patients, the activation of lung epithelial and immune cells following exposure to an allergen produces inflammatory cytokines, namely IL-4 and IL-13, which direct M2 polarization with a predominant Th2 cell response.^[16]^ This complex interplay between immune cells and products leads to a variety of changes in the airways including airway edema, bronchoconstriction, and airway remodeling, resulting in the characteristic recurrent episodes of coughing, wheezing, and shortness of breath in asthmatic patients.^[12]^

Stem cell therapy offers promising efficacy in treating inflammatory diseases such as asthma owing to its immunomodulatory and regenerative capacities.^[17]^ The anti-inflammatory effects of MSCs are manifested via cell-to-cell interactions and paracrine effects, while their potential to differentiate into multiple types of cells plays a major role in repairing damaged lung tissue.^[18,19]^ First, MSCs downregulate the activity of DCs through a mechanism involving Jagged1, a cell surface ligand implicated in the Notch signaling pathway, preventing the maturation and antigen presentation of DCs.^[17,20]^ Moreover, MSCs may suppress Th2-mediated airway inflammation by reducing type 2 cytokine production via several mechanisms: one that partly includes interferon-γ activity,^[17,21]^ and others that rely on MSC-derived EVs such as exosomes, which are suggested to have a major immunomodulatory impact in refractory asthma,^[22]^ and the recently described migrasomes, which play a pivotal role in decreasing IL-4, IL-5, and IL-13 levels.^[9]^ MSCs also regulate the inflammatory response via inducing Tregs through stem cell-T cell contact and stem cell-derived factors such as transforming growth factor-β1 and prostaglandin E2,^[23]^ especially IL-10-secreting Tregs in the lungs. Additionally, MSCs administered intravenously decrease monocyte-derived macrophage infiltration into the lungs during asthmatic inflammation and suppress M2 polarization, namely toward M2a and M2c.^[17]^

The low immunogenicity of MSCs allows them to escape the immune monitoring system. They have low levels of expression of class I and lack class II major histocompatibility complex and costimulatory molecules, giving them the advantage of crossing the immune barrier once administered. They also possess a potent migratory potential in a process called “homing,” which is a multistep process allowing them to be directed to the affected area. The low immunogenicity property, along with the homing and inflammatory chemotaxis potential of MSCs, facilitates their task in reaching the damaged tissues and exerting their immunomodulatory and regenerative capacities.^[19]^ Our asthmatic patient was administered partially differentiated bone marrow-derived stem cells both intravenously and via inhalation. The Regentime procedure’s autologous bone marrow-derived mononuclear stem cell partial differentiation enhances the active homing to the affected tissues, which are lungs in this case, after adequate incubation with tissue-specific mammal proteinic ultrafiltrate. Throughout the modern medical literature, this is the first case of complete clinical recovery of asthma signs and symptoms following autologous stem cell administration via inhalation and intravenous routes, paving the way for future studies confirming the efficacy of stem cell therapy for asthma.

Asthma can significantly impair the quality of life for some patients, requiring ongoing medical management. Our patient had complete clinical recovery of her respiratory symptoms starting 1 month after the Regentime stem cell procedure, no longer requiring previously needed asthma medications. Nevertheless, objective follow-up testing in large-scale clinical trials is essential to prove the therapeutic efficacy of stem cell therapy in asthmatic patients, establish its long-term safety, and optimize the treatment protocol to ensure maximal patient benefit.

## Acknowledgments

The authors of this manuscript are affiliated with the laboratory that supplied the ultra-filtrated peptides mentioned in the study. However, all information presented has been independently verified and any potential biases have been mitigated through rigorous fact-checking.

## Author contributions

**Conceptualization:** Rita T. Boulos, Nassim H. Abi Chahine.

**Data curation:** Rita T. Boulos, Cynthia F. Najjoum, Vanessa J. Mansour, Elsa A. El Asmar, Nassim H. Abi Chahine.

**Formal analysis:** Rita T. Boulos, Elsa A. El Asmar, Nassim H. Abi Chahine.

**Investigation:** Rita T. Boulos, Cynthia F. Najjoum, Vanessa J. Mansour, Nassim H. Abi Chahine.

**Methodology:** Rita T. Boulos, Cynthia F. Najjoum, Vanessa J. Mansour, Elsa A. El Asmar, Nassim H. Abi Chahine.

**Project administration:** Rita T. Boulos, Elsa A. El Asmar, Nassim H. Abi Chahine.

**Resources:** Rita T. Boulos, Nassim H. Abi Chahine.

**Software:** Rita T. Boulos, Cynthia F. Najjoum, Vanessa J. Mansour, Nassim H. Abi Chahine.

**Supervision:** Rita T. Boulos, Nassim H. Abi Chahine.

**Validation:** Rita T. Boulos, Nassim H. Abi Chahine.

**Visualization:** Rita T. Boulos, Nassim H. Abi Chahine.

**Writing—original draft:** Rita T. Boulos, Cynthia F. Najjoum, Vanessa J. Mansour, Elsa A. El Asmar.

**Writing—review & editing:** Rita T. Boulos, Nassim H. Abi Chahine.
